# Levels and correlates of physical activity, inactivity and body mass index among Saudi women working in office jobs in Riyadh city

**DOI:** 10.1186/s12905-016-0312-8

**Published:** 2016-06-20

**Authors:** Nada M. Albawardi, Hoda Jradi, Hazzaa M. Al-Hazzaa

**Affiliations:** Prince Sultan Center for Special Education Support Services, Po Box 75246, Riyadh, 11578 Saudi Arabia; Community and Environmental Health, College of Public Health and Health Informatics, King Saud Bin Abdulaziz University for Health Sciences, Riyadh, Saudi Arabia; Emeritus Professor and Former Director of Pediatric Exercise Physiology Research Laboratory, King Saud University, Riyadh, Saudi Arabia

**Keywords:** Physical activity, Obesity, Overweight, Saudi Arabia, Women, Office workers

## Abstract

**Background:**

Physical inactivity is among the leading risk factors for non-communicable diseases. Saudi Arabia has just begun to address physical inactivity as recent studies have shown an alarming prevalence of insufficiently physically active adults. Saudi women are identified as among the most overweight/obese and least active worldwide. With an increase in the number of women in office based jobs, the risk of physical inactivity is likely to increase. Identifying the level and correlates for high BMI and physical inactivity in Saudi women will help to plan more effective public health strategies.

The aim of this study is to assess the level of physical activity, inactivity and body mass index among Saudi women working in office based jobs in Riyadh city and identify the correlates for overweight, obesity and low physical activity.

**Methods:**

A cross- sectional study was conducted on 420 Saudi women aged 18 to 58 years working in office based jobs in eight worksites in Riyadh, Saudi Arabia. Body mass index was determined using weight and height measurements and physical activity was assessed based on a validated self-administered questionnaire.

**Results:**

The majority of the subjects were overweight or obese (58.3 %). Overweight/obesity was associated with increased age, lower income and with those working in the public versus private sector. More than half of the sample (52.1 %) were insufficiently physically active. Participants working seven or more hours per day and those working in private versus public sector were significantly associated with low physical activity.

**Conclusion:**

This study identified Saudi women working in office based jobs as a high risk group for overweight, obesity and physical inactivity. As sedentary jobs may compound the risk for obesity and physical inactivity, this may support the use of workplace health programs to reduce sitting time and promote physical activity as a viable public health initiative.

## Background

Physical inactivity is one of the major modifiable risk factors responsible for the increase in non-communicable diseases worldwide [1]. An estimated 1.9 million deaths and 19 million disability-adjusted life years are caused by physical inactivity [1]. An estimated 22 % of ischemic heart disease and 10–16 % of diabetes mellitus, breast, colon and rectal cancer are attributed to physical inactivity globally [1]. In the Kingdom of Saudi Arabia (KSA), the population attributable risk of inactivity was estimated to be 44.6 %, exceeding that of the United States (35 %) and the United Kingdom (37 %) [2].

Physical inactivity in KSA has only recently been addressed with studies showing an alarming prevalence of insufficiently physically active adults [3, 4]. In fact, 74.9 % of Saudi women were reported as being insufficiently active by the World Health Organization (WHO) in 2010, making them among the lowest group in reported prevalence of physical activity worldwide [5]. Women in KSA may be at greater risk for inactivity due to a number of factors unique to this region which includes the harsh climate, restrictions on transportation and local traditions. As the number of Saudi women entering the workforce has gradually increased in the past decade [6, 7], women employed in office based work may face additional challenges to being physically active.

Determining the prevalence of physical inactivity among Saudi women employed in sedentary jobs based on previous studies is limited. Some studies defined physical activity (PA) using only leisure time PA [3, 8] while other studies included only a small number of employed women [4, 9, 10]. The aim of this study was therefore to assess the levels and correlates of physical activity, inactivity and overweight/obesity among Saudi women working in office based jobs in Riyadh City.

## Methods

### Design and setting

A cross sectional design was used with purposeful sampling of women worksites (as gender segregated work area is the most common practice in KSA). Additional inclusion criteria included that the work is primarily office based and requiring minimal physical work. To increase generalizability, sites representing public, private and philanthropic organizations of varying sizes were identified. Selected worksites were based on the ratio of 2 to 1 for the public and private sectors, respectively, which reflects the actual ratio of employed women in the public and private sectors [6, 7]. Eleven organizations based in Riyadh, Saudi Arabia were approached for possible inclusion in the study, including academic institutions, private companies, financial institutions, government offices, and charitable organizations. Eight organizations gave approval for participating in the project and were included in the study.

### Study sample

Eligible participant had to be a female Saudi national between the age of 18 and 60 years of age and working in a primarily office based site. All available employees meeting the inclusion criteria were recruited after they were informed about the goals and significance of the study. Of the 586 eligible women approached, 420 agreed to participate in the study yielding a response rate of 72 %. Ethical approval for the study was obtained from the Institutional Review Board (IRB) at King Saud Bin Abdulaziz University for Health Sciences located at King Abdulaziz International Medical Research Center. Permission to collect data was obtained from all worksites and informed consent was obtained from all participants.

### Study instruments

#### Self-administered questionnaire

A self-administered questionnaire was used to collect demographic information and physical activity data. The physical activity questionnaire was modified from the Arab Teens Lifestyle Study (ATLS) physical activity questionnaire [11]. This instrument was found to have a high reliability and a fairly good validity against an electronic pedometer [11]. Subjects were asked to report how many days per week and for how many minutes they regularly engage in a variety of PAs, including walking as a form of exercise, walking as a means of transport, housework, moderate and vigorous activity, and stair use in the workplace and outside of work. Physical activity level was determined by calculating the ‘metabolic equivalents of task’ (MET) of each activity multiplied by minutes per week (METs-min/week), based on the compendium of PA [12]. The sum of all activities then resulted in the *total* MET*-*min/week. The subjects were then classified as low, moderate or highly active according to the classification system used by Bauman et al. [13]. The reasons for being physically active or inactive were also assessed by providing a list of possible answers in addition to an open-ended ‘other’ option.

Two psychosocial constructs that are recognized as being significantly associated with PA were assessed; general self-efficacy and social support for physical activity. Self -efficacy was evaluated using the “General Self Efficacy survey” [14]. Reliability and validity of the self- efficacy scale have been extensively tested with internal consistencies from 25 countries yielding alpha values ranging between .75–.91 [15]. The median score of the respondents was used to categorize the subject as having “high’ or “low” self- efficacy. Social support was assessed using the “Physical Activity Social Support survey” (PASS) [16]. This five item survey is a short form of the original survey developed by Sallis et al. [17] and has shown adequate validity and reliability [16]. To calculate a score for physical activity social support the questions were coded using a behavioral science scoring methodology to allow questions that are linked to be weighed appropriately. Subjects were categorized as having “high” or “low” PA social support by using the median score as a cut- off point.

The PASS and General Self Efficacy surveys were translated to Arabic, back translated to English and test-retest reliability was conducted by 15 subjects fluent in both English and Arabic completing the surveys in both languages yielding a 93.8 % agreement.

#### Anthropometric measurements

Height and weight were measured using a Seca 813 portable digital floor scale and Seca 213 portable measuring rod (Seca, Germany), respectively. Body Mass Index (BMI) (kg/m^2^) was then calculated and subjects were categorized as underweight, normal weight, overweight or obese according to the international classification used by the WHO [18].

### Data analysis

Statistical analyses were performed using SPSS version 20 (Armonk, NY). To avoid overestimation, physical activity was capped at 1680 min of PA per week (4 h of PA per day), as was used in the protocol by Bauman et al. [13] in the International Physical Activity Prevalence Study. The frequency and valid percent of respondents for each demographic variable were reported. The means and standard deviation (SD) are reported for BMI and demographic variables and the median and interquartile range (IQR) of PA (MET min/week) was also reported as it was not normally distributed. Demographic variables, BMI categories, and PA levels were then collapsed into binomial categories and differences in psychosocial and demographic characteristics among participating women and levels of BMI and PA were tested using Chi- square test with *p*-value less than 0.05 considered as significant. Bivariate logistic regression analysis was performed to identify variables associated with PA and BMI. Odds ratio (OR) and 95 % CI were obtained separately for every variable. All significant variables were then entered into a multivariate logistic regression model to adjust for confounding and identify factors associated with PA level and BMI level in this population. The reasons for being physically active or inactive were also reported and compared according to “low” and “moderate/high” level of PA groups using Chi- square test with *p*-value less than 0.05 considered as significant.

## Results

The demographic characteristics of the sample and their mean BMI and median PA levels are displayed in Tables 1 and 2 respectively. The majority of respondents (56 %) were between 26–35 years of age with a mean age of 31.7 ± 8.3. Married women constituted 45 % of the sample, while 26 % were divorced and 24 % were single. Approximately half of the sample (48 %) did not have any children. The vast majority of the respondents (85 %) had at least a college degree. The mean body mass index was 27.1 (±5.9) which lies in the ‘overweight’ BMI category. The greatest proportion of respondents were ‘normal’ weight (38.2 %); however, over fifty percent of the sample were either ‘overweight’ or ‘obese’ (58.3 %) (Table 1). The median PA METs min/week was 549.0 (IQR 181.0-1414.5) which lies in the “low” PA level category (Table 2). The majority of the respondents (52 %) reported being in the “low” PA category (<600 MET min/week) while only seven percent were in the “high” PA level category (>1500 MET min/week vigorous PA or >3000 MET min/week moderate/vigorous PA).

**Table 1:** BMI according to demographic variables

Variables	*N* (valid percent)	BMI Mean (SD)	*P* Value
Age category (*n* = 317)			
18–25	67 (21.1)	24.4 (4.85)	*P* < .001
26–35	176 (55.5)	26.7 (5.9)	
36–45	45 (14.2)	30.3 (6.2)	
46–60	29 (9.1)	31.1 (5.8)	
Marital Status (*n* = 415)			
Single	97 (23.4)	24.6 (5.2)	*P* < .001
Married	185 (44.6)	27.6 (6.0)	
Divorced	106 (25.5)	27.9 (5.4)	
Widowed	27 (6.5)	27.1 (5.9)	
Number of children (*n* = 416)			
0	200 (48.1)	25.7 (5.6)	*P* < .001
1	50 (12.0)	26.6 (6.1)	
2	44 (10.6)	27.4 (5.6)	
3	43 (10.3)	37.6 (5.0)	
4	40 (9.6)	30.6 (6.1)	
5 or more	39 (9.4)	30.4 (5.3)	
Educational Level (*n* = 416)			
Primary or less	5 (1.2)	34.7 (3.9)	*P* = .013
Middle school	7 (1.7)	28.3 (5.7)	
High school	53 (12.7)	28.4 (6.0)	
College diploma/bachelor	334 (80.4)	26.7 (5.8)	
Postgraduate degree	17 (4.1)	27.1 (5.9)	
Monthly family income			
(Saudi Riyals) (*n* = 392)			
5000 or less	35 (8.9)	28.6 (6.1)	*P* = .178
5001–7000	69 (17.6)	26.7 (6.5)	
7001–10,000	64 (16.3)	28.5 (7.5)	
10,001–15,000	74 (18.9)	26.4 (4.3)	
15,000–20,000	56 (14.3)	26.4 (5.3)	
Over 20,000	94 (24.0)	27.1 (5.5)	
Size of home (*n* = 409)			
Traditional (folk)	4 (1.0)	30.5 (6.2)	*P* = .281
Apartment	93 (22.7)	27.4 (6.7)	
Small villa (<500 m^2^)	95 (23.2)	27.8 (5.4)	
Medium (500–1000 m^2^)	175 (42.8)	26.4 (5.8)	
Large (over 1000 m^2^)	42 (10.3)	27.3 (5.4)	
Home ownership (*n* = 413)			
Rental	109 (26.4)	26.7 (5.4)	*P* = .004
Owned	297 (71.9)	27.1 (5.9)	
Employer provided	7 (1.7)	34.8 (7.1)	
Number of work days/week (*n* = 419)			
5	412 (98.1)	27.0 (5.8)	*P* = .669
6	7 (1.7)	28.0 (6.1)	
Number of working hours/day (*n* = 412)			
1–4 h	15 (3.6)	27.6 (6.7)	*P* = .970
5–6 h	134 (32.4)	27.3 (5.7)	
7–8 h	197 (47.7)	27.0 (6.0)	
9–10 h	60 (14.5)	26.7 (5.9)	
Over 10 h	6 (1.5)	26.7 (4.3)	
Job description (*n* = 399)			
Supervisor	121 (28.8)	27.1 (5.9)	P = .777
Non supervisor	278 (66.2)	27.1 (5.9)	
Job Sector (*n* = 402)			
Public	262 (65.2)	28.0 (5.9)	*P* < .001
Private	140 (34.8)	25.3 (5.4)	
Physical Activity Social Support score (*n* = 404)			
Low	194 (48.0)	26.8 (5.6)	*P* = .386
High	210 (52.0)	27.3 (6.1)	
General Self-Efficacy score (*n* = 417)			
Low	169 (41.0)	26.8 (5.7)	*P* = .447
High	248 (59.0)	27.2 (5.9)	
BMI category^a^ (*n* = 393)			
Underweight	14 (3.6)	17.0 (1.3)	*P* < .001
Normal weight	150 (38.2)	22.5 (1.7)	
Overweight	127 (32.3)	27.3 (1.3)	
Obese	102 (26.0)	34.8 (4.6)	
Total	393 (100)	27.1 (5.9)	
PA level^b^ (*n* = 420)			
Low	219 (52.1)	26.9 (6.0)	*P* = .822
Moderate	173 (41.2)	27.3 (5.8)	
High	28 (6.7)	26.9 (4.8)	

^a^Underweight: <18.5; normal: 18.50–24.99; overweight: 25.00–29.99; obese: ≥30.0

^b^Low: <600MET min/week; moderate: 600–2999 MET min/week; high: ≥1500 MET min/week vigorous PA or ≥3000 MET min/week moderate/vigorous PA

**Table 2:** PA level according to demographic variables

Variables	*N* (valid percent)	PA level (MET-min/week)		*P* Value
		Mean (SD)	Median (IQR)	
Age category (*n* = 317)				
18–25	67 (21.1)	1006.0 (1242.6)	466.7 (136.7–1460.7)	*P* = .984
26–35	176 (55.5)	1041.6 (1195.7)	581.0 (184.6–1550.8)	
36–45	45 (14.2)	1092.0 (1140.2)	658.7 (308.3–1517.3)	
46–60	29 (9.1)	1015.5 (788.0)	784.0 (310.3–1667.3)	
Marital Status (*n* = 415)				
Single	97 (23.4)	867.4 (1145.9)	416.0 (141.8–1104.3)	*P* = .260
Married	185 (44.6)	1063.8 (1193.5)	580.0 (182.0–1662.0)	
Divorced	106 (25.5)	1039.0 (1072.4)	733.7 (318.2–1288.6)	
Widowed	27 (6.5)	685.8 (951.2)	270.0 (101.3–866.0)	
Number of children (*n* = 416)				
0	200 (48.1)	869.7 (1085.4)	434.7 (149.3–1255.0)	*P* = .054
1	50 (12.0)	988.3 (993.8)	620.7 (252.3–1486.6)	
2	44 (10.6)	1443.4 (1415.0)	1073.7 (187.7–2477.3)	
3	43 (10.3)	902.3 (1170.2)	453.3 (156.0–1244.7)	
4	40 (9.6)	1192.4 (1282.2)	676.7 (332.4–1683.8)	
5 or more	39 (9.4)	926.4 (953.2)	641.3 (186.7–1440.0)	
Educational Level (*n* = 416)				
Primary or less	5 (1.2)	1098.8 (1136.7)	369.7 (253.8–2308.3)	*P* = .033
Middle school	7 (1.7)	1243.0 (1188.9)	941.3 (58.7–2150.0)	
High school	53 (12.7)	1440.1 (1515.9)	1067.3 (261.5–1923.7)	
College diploma/bachelor	334 (80.4)	917.0 (1067.3)	532.7 (173.2–1315.7)	
Postgraduate degree	17 (4.1)	822.4 (874.3)	553.3 (114.7–1286.7)	
Monthly family income (Saudi Riyals) (*n* = 392)				
5000 or less	35 (8.9)	1420.5 (1477.5)	1027.3 (257.0–2150.0)	*P* = .179
5001–7000	69 (17.6)	785.9 (864.0)	456.0 (166.5–1149.8)	
7001–10,000	64 (16.3)	906.9 (1252.9)	410.0 (130.8–1061.7)	
10,001–15,000	74 (18.9)	1055.8 (1200.5)	704.0 (217.7–1438.3)	
15,000–20,000	56 (14.3)	1004.3 (959.3)	615.7 (212.3–1722.4)	
Over 20,000	94 (24.0)	1028.4 (1203.9)	552.3 (177.2–1393.0)	
Size of home (*n* = 409)				
Traditional (folk)	4 (1.0)	1794.0 (2406.2)	827.0 (226.7–4328.4)	*P* = .117
Apartment	93 (22.7)	1204.0 (1347.6)	710.7 (189.2–1597.3)	
Small villa (<500 m^2^)	95 (23.2)	928.0 (990.5)	590.7 (200.0–1470.7)	
Medium (500–1000 m^2^)	175 (42.8)	962.3 (1114.0)	525.3 (185.7–1309.3)	
Large (over 1000 m^2^)	42 (10.3)	753.8 (896.0)	404.0 (131.2–1006.1)	
Home ownership (*n* = 413)				
Rental	109 (26.4)	1193.8 (1283.5)	705.3 (249.7–1607.7)	*P* = .062
Owned	297 (71.9)	905.0 (1059.5)	480.0 (173.3–1270.8)	
Employer provided	7 (1.7)	1272.2 (1802.3)	710.7 (208.0–1101.3)	
Number of work days/week (*n* = 419)				
5	412 (98.1)	979.6 (1131.0)	552.3 (184.0–1414.5)	*P* = .700
6	7 (1.7)	1146.6 (1526.4)	287.7 (58.7–2640.0)	
Number of working hours/day (*n* = 412)				
1–4 h	15 (3.6)	1043.3 (763.7)	1001.7 (320.7–1609.7)	*P* = .022
5–6 h	134 (32.4)	1212.9 (1205.5)	715.0 (370.0–1722.0)	
7–8 h	197 (47.7)	852.8 (1102.9)	408.0 (140.0–1168.0)	
9–10 h	60 (14.5)	782.3 (996.0)	332.3 (113.3–1088.6)	
Over 10 h	6 (1.5)	1527.6 (1856.7)	775.3 (364.7–2722.7)	
Job description (*n* = 399)				
Supervisor	121 (28.8)	1129.7 (1182.7)	699.3 (211.8–1709.0)	*P* = .144
Non supervisor	278 (66.2)	945.9 (1139.9)	510.7 (183.0–1317.2)	
Job Sector (*n* = 402)				
Public	262 (65.2)	1119.0 (1183.6)	681.7 (261.2–1563.6)	*P* < .001
Private	140 (34.8)	650.2 (935.4)	254.7 (112.0–811.2)	
Physical Activity Social Support score (*n* = 404)				
Low	194 (48.0)	939.2 (1162.8)	475 (177.2–1268.3)	*P* = .314
High	210 (52.0)	1054.6 (1136.4)	648 (190.7–1559.6)	
General Self-Efficacy score (*n* = 417)				
Low	169 (41.0)	1037.5 (1263.2)	525 (165.5–446.7)	*P* = .413
High	248 (59.0)	944.6 (1043.5)	571 (186.7–1383.8)	
BMIª category (*n* = 393)				
Underweight	14 (3.6)	677.3 (1037.1)	288.5 (72.7–915.0)	*P* = .671
Normal weight	150 (38.2)	971.3 (1125.7)	531.5 (186.6–1410.2)	
Overweight	127 (32.3)	1043.9 (1176.7)	677.3 (186.7–1440.0)	
Obese	102 (26.0)	942.8 (1053.1)	539.2 (169.6–1498.7)	
PA^b^ level (*n* = 420)				
Low	219 (52.1)	229.1 (164.3)	186.7 (90.67–337.3)	*P* < .001
Moderate	173 (41.2)	1411.8 (644.1)	1309.3 (832.665–1882.7)	
High	28 (6.7)	4185.2 (86.7)	4078.0 (3260.75–5101.6)	
Total	420 (100)	980.0 (1136.2)	549.0 (181.0–1414.5)	

^a^Underweight: <18.5; normal: 18.50–24.99; overweight: 25.00–29.99; obese: ≥30.0

^b^Low: <600MET min/week; moderate: 600–2999 MET min/week; high: ≥1500 MET min/week vigorous PA or ≥3000 MET min/week moderate/vigorous PA

The subjects’ psychosocial variables included their perceived social support for physical activity from family and friends. Using the composite median score of 11.0 (IQR 6.0-16.0) to categorize subjects as having “high” or “low” social support ; 48.0 % of respondents were then considered as having “low physical activity social support” (Table 1).

The second psychosocial variable was general self- efficacy. The average score on the ten items resulted in a median of 3.0 (IQR 2.8-3.3) resulting in 59.0 % of the subjects being categorized as having “high self – efficacy” (Table 1).

### Factors associated with being overweight or obese

Bivariate analysis showed a significantly greater proportion of respondents were ‘overweight or obese’ if they were over 35 years old (*p* < 0.001), married (*p* = 0.047), had at least one child (*p* = 0.001), had an education level above high school (*p* = 0.008), a family income of less than 10,000 Saudi Riyals (SR) (*p* = 0.019) and worked in the public sector (*p* < 0.001) (Table 3). Physical activity social support was not found to be significantly associated with being overweight or obese in this sample (*p* = .678) (Table 3). No significant relationship was found between being overweight or obese and level of self-efficacy (*p* = . 656) (Table 3).

**Table 3:** Proportion of respondents with normal versus overweight/obese BMI according to demographic groups

Respondent characteristics	BMI category		*p*-value
	Normal	Overweight/Obese	
	*N* (%)	*N* (%)	
All (*n* = 393)	150 (38.2)	229 (58.3)	----
Age (*n* = 283)			
≤ 35 years	103 (36.4)	112 (39.6)	*P* < 0.001
> 35 years	12 (4.2)	56 (19.8)	
Marital status (*n* = 415)			
Married	56 (15.0)	108 (28.9)	*P* = 0.047
Other (not married)	93 (24.9)	117 (31.3)	
Number of children (*n* = 378)			
none	87 (23.0)	92 (24.3)	*P* = 0.001
At least one	63 (16.7)	136 (36.0)	
Education (*n* = 375)			
≤high school	15 (4.0)	46 (12.3)	*P* = 0.008
> high school	134 (35.7)	180 (48.0)	
Monthly family income (*n* = 353)			
< 10,000 SR (2,667 USD)	48 (13.6)	102 (28.9)	*P* = 0.019
≥10,0000 SR (2,667 USD)	90 (25.5)	113 (32.0)	
Job sector (*n* = 361)			
Public	77 (21.3)	162 (44.9)	*P* < 0.001
Private	66 (18.3)	56 (15.5)	
Working hours per day (*n* = 371)			
< 7 h	56 (15.1)	84 (22.6)	*P* = 0.974
≥7 h	92 (24.8)	139 (37.5)	
Physical Activity Social Support score (*n* = 366)			
Low	70 (19.1)	103 (28.1)	*P* = 0.678
High	74 (20.2)	119 (32.5)	
Self-Efficacy score (*n* = 377)			
Low	58 (15.4)	94 (24.9)	*P* = 0.656
High	91 (24.1)	134 (35.5)	
PA level (*n* = 379)			
Low	78 (20.6)	116 (30.6)	*P* = 0.798
Moderate/high	72 (19.0)	113 (29.8)	

Findings from the multivariate logistic regression analysis are shown in Table 4. Age appears to increase risk of overweight or obesity by an OR of 1.1 (95 % CI 1.06–1.54). Those with a monthly family income of less than 10,000 SR (2,667 USD) (were two times as likely to be overweight or obese than those with a higher income (95 % CI 1.23–3.87), while working in the public sector versus the private sector increased risk for overweight and obesity by an OR of 1.78 (95 % CI 1.0–3.17).

**Table 4:** Results of logistic regression of variables predictive of overweight or obesity

Variable	OR	95 % CI	Wald	*P* value
Age	1.1	1.06–1.54	19.1	<0.001
Family income <10,000 SR (2,667 USD)	2.19	1.23–3.87	7.19	0.007
Work in public sector	1.78	1.0–3.17	3.83	0.050

### Factors associated with low physical activity level

Bivariate analysis showed a greater proportion of respondents had low level of physical activity if they worked seven or more hours per day (*p* = 0.001), did not have children (*p* = 0.032) and worked in the private sector (*p* < 0.001) (Table 5). Level of physical activity social support was not significantly associated with PA level (*p* = .130) (Table 5). Similarly no significant relationship was found between PA level and self-efficacy (*p* = .542) (Table 5).

**Table 5:** Proportion of respondents with low versus moderate/high PA level according to demographic groups

Respondent characteristics	Low physical activity level	Moderate/high physical activity level	*p*-value
	*N* (%)	*N* (%)	
All	219 (52.1)		
Age (*n* = 317)			
≤ 35 years	126 (39.7)	117 (36.9)	*P* = 0 .195
> 35 years	32 (10.1)	42 (13.2)	
Marital status (*n* = 415)			
Married	97 (52.4)	88 (47.6)	*P* = 0.819
Other (not married)	118 (51.3)	112 (48.7)	
Number of children (*n* = 416)			
None	115 (57.5)	85 (42.5)	*P* = 0.032
At least one	103 (47.0)	116 (53.0)	
Education (*n* = 416)			
≤high school	27 (41.5)	38 (58.5)	*P* = 0.068
> high school	189 (53.8)	162 (46.2)	
Monthly family income (*n* = 392)			
< 10,000 SR (2,667 USD)	91 (54.2)	77 (45.8)	*P* = 0.414
≥ 10,0000 SR (2,667 USD)	112 (50.0)	112 (50.0)	
Job sector (*n* = 402)			
Public	118 (45.0)	144 (55.0)	*P* < 0.001
Private	94 (67.1)	46 (32.9)	
Working hours per day (*n* = 412)			
< 7 h	61 (40.9)	88 (59.1)	*P* = 0.001
≥ 7 h	154 (58.6)	109 (41.4)	
Physical Activity Social Support score (*n* = 366)			
Low	107 (55.2)	87 (44.8)	*P* = 0.130
High	100 (47.6)	110 (52.4)	
Self-Efficacy score (*n* = 417)			
Low	91 (53.8)	78 (46.2)	*P* = 0.542
High	126 (5.8)	122 (49.2)	
BMI (*n* = 393)			
Normal	78 (52.0)	72 (48.0)	*P* = 0.798
Overweight/obese	116 (50.7)	113 (49.3)	

Multivariate logistic regression analysis of these variables showed two factors were predictive of low PA; those working seven or more hours per day had an OR of 1.67 (95 % CI 1.07–2.61) for low PA level while those in the private sector were over two times more likely to be insufficiently active (95 % CI 1.32–3.33) (Table 6).

**Table 6:** Results of logistic regression of variables predictive of low physical activity level

Variable	OR	95 % CI	Wald	*P* value
Working ≥7 h/day	1.67	1.07–2.61	5.05	0.025
Work in private sector	2.1	1.32–3.33	9.80	0.002

The reasons participants with “low or “moderate/high” reported PA gave for being active or inactive found all participants reported ‘health reasons’ as the most important reason for being active while ‘maintaining their weight’ was second but significantly more important for the higher PA group (*p* = .027) (Table 7). Both the lower and higher PA groups reported ‘not having time’ and being ‘too tired’ as the most frequent reasons for not being PA, however a significantly greater number of respondents in the low PA group (*p* = .009) reported that ‘not having time’ was the main obstacle for being active. Physical activity being ‘socially unacceptable’ was the least frequently mentioned reason for being inactive by both groups.

**Table 7:** Reasons given by Saudi employed women who have low or high physical activity for being physically active or inactive (more than one answer could be chosen)

Reasons for being physically active (*n* = 417)	Low PA *N* (%)	High PA *N* (%)	*P*-value
For health	112 (54.1)	130 (61.9)	.107
To maintain bodyweight	86 (41.5)	110 (52.4)	.027
To reduce body weight	99 (47.8)	97 (46.2)	.738
For recreation	76 (36.7)	75 (35.7)	.832
To spend time with friends	4 (1.9)	10 (4.8)	.109
For competition	1 (0.5)	4 (1.9)	.182
Other reasons for being PA	11 (5.3)	11 (5.2)	.972
Reasons for not being physically active (*n* = 418)			
No time	148 (71.2)	124 (59.0)	.009
Too tired to exercise	93 (44.7)	80 (38.1)	.170
Facilities are too far	65 (31.2)	57 (27.1)	.356
No transportation	46 (22.1)	48 (22.9)	.856
No one to exercise with	50 (24.0)	40 (19.0)	.215
No will power	39 (18.8)	28 (13.3)	.131
No motivation	12 (5.8)	4 (1.9)	.039
Facilities are too expensive	31 (14.9)	30 (14.3)	.858
Due to health reasons	19 (9.1)	19 (9.0)	.975
Don’t know how to exercise/play sports	10 (4.8)	10 (4.8)	.983
Due to health reasons	19 (9.1)	19 (9.0)	.975
Don’t know how to exercise/play sports	10 (4.8)	10 (4.8)	.983
Socially unacceptable	5 (2.4)	4 (1.9)	.725
Other reasons for not being PA	14 (6.7)	11 (5.2)	.499

## Discussion

Prevalence of obesity in this sample of women working in office settings (26 %) was lower than the 43 % previously reported by the WHO [5] for women in the KSA. It also fell below the 44 % of women reported as obese in a large (*n* = 17,232) cross sectional household survey conducted between 1995–2000 [19]. Another household survey in 2013 (*n* = 10,735) also reported obesity prevalence among women as 33.5 %, greater than the present sample [20]. The variation in prevalence from these and the present study may be the result of the large cross sectional studies including subjects from different cities in the KSA and from rural areas which are known to differ in their BMI. Education level, a significant predictor of obesity [21], also differed between these studies with one study [20] reporting only 21 % of participants having a college degree or higher while in the present study it was 80 %. The varying employment status of the subjects may also affect obesity levels as it has been reported that a greater proportion of unemployed women in the KSA are obese as compared to employed women [21]. It is therefore difficult to compare the prevalence of the present study with those reported previously due to the difference in the characteristics of the study groups.

In the present sample, participants had a significantly greater risk of overweight and obesity when family income was less than 10,000 Saudi Riyals (2,667 USD). In fact it was one of only three predictors for high BMI. A positive association between socioeconomic status (SES) and BMI and between SES and overweight has been demonstrated in a sample of over 500,000 women from 54 low to middle-income countries [22]. In developing countries, the burden of obesity tends to shift towards the groups with lower SES as the country’s gross national product improves. This shift occurs at an earlier stage of economic development for women than it does for men [23] resulting in a particularly greater danger for women. Whether the association found in this study is only in employed Saudi women or exists in the general public is unclear and warrants further research.

The greater proportion of overweight and obese women over the age of 35 years, married and having children is to be expected [24, 25], however it is alarming to find that 44 % of women 18 to 25 years of age were overweight or obese which is greater than has been previously reported [24]. Over a third of single women and 51.4 % of women without children were also overweight or obese. As a number of these women are expected to marry and bear children, their risk for increased body weight will also increase along with a higher chance for the co morbidities of obesity.

Working in the public sector was also found to be a predictor of overweight/obese independent of other variables (Table 4). No studies were found on this subject however as most government workers in the KSA have tenure, it may be attributed to a more relaxed culture in government workplaces which were also observed to be more flexible about the presence of food in work areas. Government workers may also be under less social pressure to present a fit body image to clients as compared to those working in the private sector. Further investigation into other variables in the workplace such as availability and types of food consumed and social factors is warranted.

The total physical activity level reported by this sample showed that 52.1 % of the respondents were insufficiently active, exceeding previous global and local reports [4, 13, 26]. Hallal et al. [26] reported on PA levels in adults from 122 countries and found 31 % to be physically inactive (range 17–43 %). Bauman et al. [13], in a comparative international study of population PA prevalence, used a self-report survey, the International Physical Activity Questionnaire (IPAQ), across 20 countries and reported low PA levels in females ranging from 6 to 49 %. The IPAQ has been shown to over report physical activity [27] yet Saudi Arabia participated in this study and reported only 37.3 % of their female sample (*n* = 344) as having low level PA. The occupational status of that sample is unreported and only 38.3 % of all the Saudi participants in that study had an education level above high school unlike the present sample which exceeded 80 %. As higher educational level is associated with more sedentary professions, this may be an important variable explaining the greater prevalence of inactivity in the present study. Al-Hazzaa [4] in a sample including 365 adult females in Riyadh assessed PA using the short form IPAQ and found 34.3 % to be in the lowest PA level, however less than 20 % were employed. The sample was also older, the majority (44 %) being 30–44 years, while the present study was predominately 26–35 years (55 %). Amin et al. (2012) [28] reported on a sample from primary care centers in AlHassa (*n* = 967) using a survey derived from the IPAQ and found approximately 80 % of respondents were insufficiently active. Only a third of the females in his sample were employed and less than 40 % had over high school level education levels.

Predictors of low physical activity included ‘working seven or more hours per day ’ (Table 6) and it is reasonable to assume that the long working hours in office based jobs would reduce the time spent in PA. This is consistent with the subjects reporting “not having enough time” as the main reason for not being active (Table 7). Longer working hours, particularly in sustained postures are known to cause fatigue even when physical exertion has not been expended [29] and may also explain the second highest reported barrier for PA being “too tired” (Table 7).

Working in the private sector was a second predictor of low physical activity independent of work hours (Table 6). This merits further investigation however the private sector in the KSA is known to have a more competitive work environment and greater work load compared to public sector jobs which offer early tenure and tend to have more employees for similar work loads. This may contribute to more sitting and greater fatigue after work for private sector employees.

Surprisingly psychosocial variables often found to correlate with PA, self-efficacy and social support, were not found to be significant in this sample. Social support was found to be an important variable in one previous study in KSA [8] and in numerous international studies [30–32]. In this study however no correlation was found between physical activity social support scores and BMI or PA. In addition, only 21.5 % of respondents mentioned “no one to exercise with” as a factor for their inactivity. Self- efficacy, which has been found to correlate to PA in the majority of international literature [32–34] and in one study on women in KSA [10], was not significantly correlated to PA in this sample. Major barriers to PA noted by both low and highly active respondents; ‘not having time’ and being ‘too tired’ may be interpreted as a lack of self- efficacy and it is possible that using a survey targeting *physical activity* self-efficacy may have been more sensitive to differences among respondents than the ‘general self- efficacy’ survey used in this study.

Environmental factors including ‘distance to facilities’, ‘lack of transportation’ and ‘facilities being too expensive’ were barriers to activity for less than 30 % of this sample and PA being ‘socially unacceptable’ was mentioned by the least number of respondents as a reason for inactivity (2 %) (Table 7). This is in contrast to previous studies in KSA [8, 9] which reported lack of facilities/resources and traditions as the most important barriers. This may be explained by the present study sample being predominately educated professionals whose awareness of the importance of physical activity may override local traditions. Their professions may also allow them more access to facilities than the predominately unemployed samples used in the previous studies.

Interestingly there was no significant difference in the proportion of normal weight and overweight/obese respondents reporting low PA level in this sample which is similar to previous results on female Saudi university students [35].

This study was subject to several limitations including the use of a survey as the main study instrument which is subject to recall bias and social desirability effect. Therefore, despite using a reliable and valid physical activity questionnaire in this survey, it is recommended that future studies may consider the use of more objectives instruments for the assessments of physical activity in Saudi working women, such as accelerometer. Secondly, the cross sectional design limits the ability to make causal inferences. Another limitation is that the sample was from Riyadh city which decreases generalizability of the findings to other regions in the country.

## Conclusion

This study identified Saudi women working in office based jobs as a high risk group for overweight, obesity and physical inactivity. The finding that increasing age was a predictor of higher BMI was to be expected, however, the high percentage (44 %) of young Saudi females (18–25 year olds) who were found to be overweight or obese, projects for a possible high future obesity trend among women in KSA. Reports of low physical activity exceeded global and local reports and were correlated to long working hours and working in the private sector. As time constraints and fatigue were the most often reported barriers for PA, this may support the use of workplace health programs to reduce sitting time and promote physical activity as a viable public health initiative.

## Abbreviations

ATLS, Arab Teens Lifestyle Study; BMI, body mass index; IPAQ, International Physical Activity Questionnaire; IQR, inter quartile range; KSA, Kingdom of Saudi Arabia; MET, metabolic equivalents of task; OR, odds ratio; PA, physical activity; PASS, Physical Activity Social Support; SD, standard deviation; SR, Saudi riyal; USD, United States Dollar; WHO, World Health Organization
